# Comparison of histopathological and triphenyl tetrazolium chloride test in diagnosing myocardial infarction: An autopsy study

**DOI:** 10.12688/f1000research.152421.1

**Published:** 2024-09-12

**Authors:** Varun Krishna B, Chandni Gupta, Vikram Palimar, Anitha S, Deepak Nayak M

**Affiliations:** 1 Department of Forensic Medicine and Toxicology, Ganapathichettikulam Village No. 20, Pondicherry Institute of Medical Sciences, Kalapet, Puducherry, 605014, India; 2 Department of Anatomy, Kasturba Medical College, Manipal Academy of Higher Education, Manipal, Manipal, Karnataka, 576104, India; 3 Department of Forensic Medicine and Toxicology, Kasturba Medical College, Manipal, Manipal Academy of Higher Education, Manipal, Karnataka, 576104, India; 4 Department of Pathology, Kasturba Medical College, Manipal, Manipal Academy of Higher Education, Manipal, Karnataka, 576104, India

**Keywords:** TTC (Triphenyl Tetrazolium Chloride) staining, histopathological examination, Myocardial infarction, Autopsy, Cardiovascular diseases.

## Abstract

**Background:**

Sudden and unexpected deaths are increasing drastically. The main cause of sudden death is cardiovascular disease, out of which coronary artery disease predominates forming 80% of the cases. Most of the time, detecting early changes in myocardial infarction during the autopsy is challenging since gross infarct changes do not appear until after 24 to 48 hours of myocardial ischemia injury. So, the aim of this study was to compare two test to detect early changes of Myocardial Infarction one by using Triphenyl Tetrazolium Chloride (TTC) staining of the myocardial tissue, during autopsy and other by histopathological examination.

**Methods:**

The sample size of 60 hearts taken from all the sudden deaths cases brought to Mortuary with suspected cause of death due to cardiac origin. The heart was obtained from the deceased by standard post-mortem technique. Serial full-thickness transverse sections of the heart were taken at 2 cm intervals from the apex to the atrioventricular groove. All the serial slices of heart are taken for histochemical staining and TTC staining.

**Results:**

In histopathological examination 34 hearts were diagnosed with myocardial infarction and 26 hearts reported non myocardial infarction. With TTC 40 hearts remained unstained suggestive of myocardial infarction and 20 hearts were stained suggestive of non-infarcted hearts. TTC staining in our study shows an accuracy of 88.33%.

**Conclusion:**

The result of this study shows that the Triphenyl Tetrazolium Chloride test, a histochemical staining technique of heart, is reliable approach for forensic pathologists to diagnose early myocardial infarction during the post-mortem examination.

## Introduction

Globally, sudden, and unexpected deaths are increasing drastically. The main cause of sudden death is cardiovascular disease, out of which coronary artery disease predominates forming 80% of the cases. In 25% of the cases death occurred suddenly and in an unexpected manner within an hour of inception of clinical signs. Myocardial infarction acts as key psychological and legal consequences to both society and individual. ^1^

As most deaths are abrupt, establishing a clinical diagnosis of Myocardial Infarction can be challenging. Postmortem examination is the ultimate chance to opine the cause of death in such cases. In any Medico-Legal Autopsy, the goal is to determine the cause of death. Most of the time, detecting early changes in myocardial infarction during the autopsy is challenging since gross infarct changes do not appear until after 24 to 48 hours of myocardial ischemia injury. Hence the determination of the early stage of myocardial ischemia is still a difficult task. ^2^

The use of azo dyes to stain the heart helps to distinguish infarcted regions from normal myocardium. According to histochemical staining techniques, the membrane integrity of ischemic myocardial cell is lost, and enzymes are released into blood circulation, which results in reduction or complete absence of these enzymes in those regions. The rationale behind this study is that enzyme-depleted infarct myocardium will not be stained. ^3^

So, the aim of this study was to compare two test to detect early changes of Myocardial Infarction one by using Triphenyl Tetrazolium Chloride (TTC) staining of the myocardial tissue, when there are no gross macroscopic changes, during autopsy and other by histopathological examination in the unstained regions of myocardium. This study also investigated how accurate histochemical Staining (TTC) of the heart is at detecting early myocardial infarction.

## Methods

Study type: Observation study.

Kasturba Medical College and Kasturba Hospital Institutional ethical clearance was taken before starting the study. Approval no with date: 705/2019, 17.9.2019.

We used the STROBE reporting guidelines for our study; a completed checklist is available under Reporting Guidelines. ^4^

Study Period: The current research was carried out for two years.

Study location: Department of Forensic Medicine in association with the Department of Pathology.

Sample Size: 60 hearts.

Inclusion Criteria: Heart was taken out from all the sudden deaths cases brought to Mortuary with suspected cause of death due to cardiac origin and without any foul play and this was corroborated with the history stated in Karnataka police inquest 146 (i) and (ii).

Exclusion criteria: Death due to other reason like suicide, hanging and drowning etc were excluded from the study.

### Detailed description of procedure/processes

Ethics committee has given the exemption from taking consent from the relatives of the deceased since these cases are postmortem cases where the law gives the consent for autopsy and determination of cause of death. In the above cases the samples were taken from death due to cardiac origin so, the heart must be taken out to determine the cause of death. The consent given by the law for autopsy is valid. The heart was obtained from the deceased by standard post-mortem technique. The heart was thoroughly washed under running water and weight of the heart is noted. The entire heart was examined grossly for any pathological abnormalities, scarring due to old infarctions, areas of hyperemia, and external injuries. The serial cut sections of all three coronary arteries were made at the distance of 3 mm starting from the origin and throughout its course to look for the existence of blockage of its lumen by plaques or thrombus. The texture of coronaries was noted. Serialized full-thickness transverse slices of the heart were taken at 2 cm intervals from the apex to the atrioventricular groove. The sliced parts are inspected for fibrotic scarring and myocardial softening. The heart was dissected all along the line of blood flow (in flow and out flow procedure) and examined for atheromatous plaques on the surface of the origin of the aorta, narrowing of the coronary ostia, and the walls and valves. All the serial slices of heart are taken for histochemical staining.”

### Preparation of TTC solution

“The following chemicals are needed which were procured from Durga, Laboratory, Mangalore, Karnataka:

1) NaH2Po4 (Sodium Dihydrogen Phosphate) - phosphate buffer with low pH. (Molecular weight 120 g)
2) Disodium hydrogen phosphate (Na2HPo4) - phosphate buffer with high pH (Molecular weight being 142 g)
3) Distilled water. (1litre)
4) Triphenyl Tetrazolium Chloride dye (TTC) (10grams)

A 0.1 M solution was made by mixing 12 g of Sodium Dihydrogen phosphate in one litre of distilled water. Similarly, a 0.1 M solution was made by dissolving 14.2 g of sodium hydrogen phosphate in one litre of distilled water. ^5^ The above two solutions were then mixed in different proportions to adjust the pH and is shown in Table 1.

**Table 1.** Showing the two solutions mixed in different proportions to adjust the pH.

pH	0.1 M Na2HPO4 Solution, (High pH)	01 M Na2H2P04 Solution, Low pH (%)
5.8	079 ml	921 ml
6.0	120 ml	880 ml
6.2	178 ml	822 ml
6.4	255 ml	745 ml
6.6	355 ml	645 ml
6.8	463 ml	537 ml
7.0	577 ml	423 ml
7.2	684 ml	316 ml
7.4	774 ml	226 ml
7.6	845 ml	155 ml
7.8	896 ml	104 ml
8.0	932 ml	68 ml

To achieve a pH of 7.8“896 ml of 0.1M sodium Dihydrogen phosphate solution and 104 ml of 0.1 M Disodium hydrogen phosphate solution were combined. A pH meter was used to confirm the pH of the buffer solution. To prepare a 1% TTC solution, ten grams of Triphenyl Tetrazolium Chloride were mixed in 1 litre of above phosphate buffer solution with a pH of 7.8. ^5^ Because TTC salt is photosensitive and inactivates when exposed to light, it was stored in an Amber colored bottle.

### Staining method

The multiple serial sections of the heart were rinsed under running water and mopped with clean cloth and positioned in a glass jar. To avoid improper staining technique precautions were taken for overlaying each heart slice and it was stained in a separate glass container. To prevent ambient oxygen from entering the heart sections, TTC solution was then filled in each container containing heart sections to a level of 2 cm above the heart section. To avoid light exposure, the container is then placed in a dark room. The incubation period of 30 minutes is carried out at room temperature. After incubation, heart sections were taken out from the solution and kept in 10 % formalin solution to end the procedure of staining and helps in fixation. Then it was examined for unstained areas in TTC positive cases for the myocardium suggestive of myocardial infarction. Normal myocardium appears brick red or bossy red because of lactate dehydrogenase activity in normal myocardial tissues. The infarcted area remained unstained with the TTC in the positive outcome. The infarct area, on the other hand, remains unstained or pale in color. Paraffin embedded histological microscopic examination was performed on both the stained and unstained areas. From unstained regions, the sections are subjected for histopathological examination and smeared with Hematoxylin-Eosin for microscopic diagnosis of acute myocardial infarction. The gross examination of heart results of TTC staining was then confirmed with histological findings.

### Statistical analysis

Descriptive analysis was done. Prevalence was also noted. Results were also analysed in percentage.

Underlying data is included in the Underlying Data section. ^6^

## Results

The distribution of gender and age in the study sample is shown in Table 2. 71.7% of cases belong to the age group 31 to 60 years, 18.3% cases are in the age group more than 60 years. less than 30 years account for 10% of total cases. Majority of the cases belongs to males (91.7%) whereas female was only 8.3% of the cases. This indicates that death due to myocardial infarction occurs mostly in males.

**Table 2.** Distribution of gender and age in study sample.

Age category	Gender		Total
	Male	Female	
<30 years	6	0	6
	100.0%	0.0%	100.0%
31-60 years	40	3	43
	93.0%	7.0%	100.0%
>61 years	9	2	11
	81.8%	18.2%	100.0%
Total	55	5	60
	91.7%	8.3%	100.0%

### Histopathological examination

**Figure 1.**
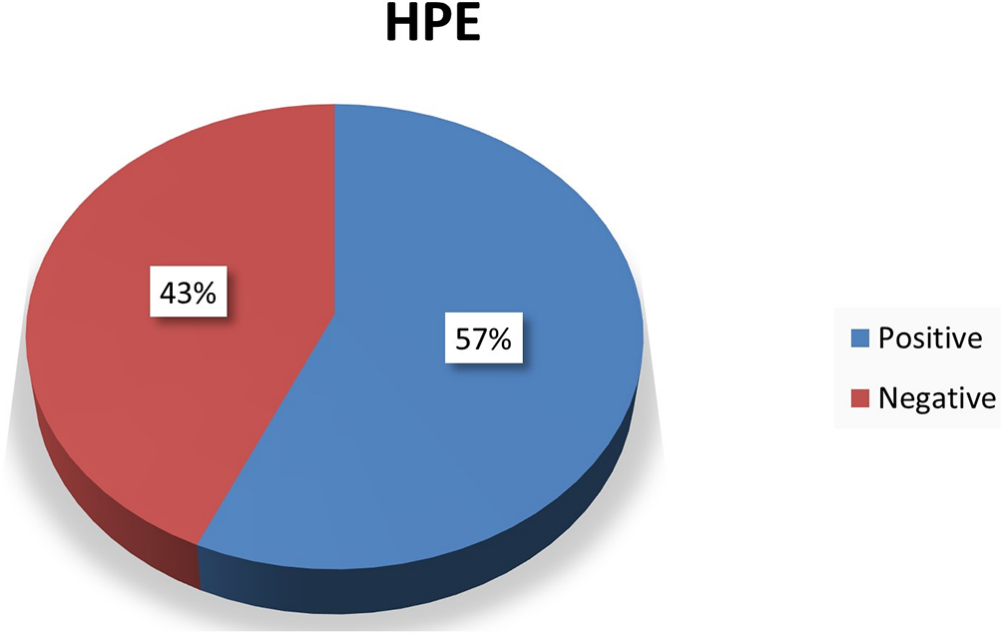
Chart showing histopathological examination results.

60 hearts were subjected for histopathological examination out of which 34 hearts were diagnosed with myocardial infarction and 26 hearts reported non myocardial infarction ( Figure1).

### Triphenyl Tetrazolium Chloride (TTC) staining

**Figure 2.**
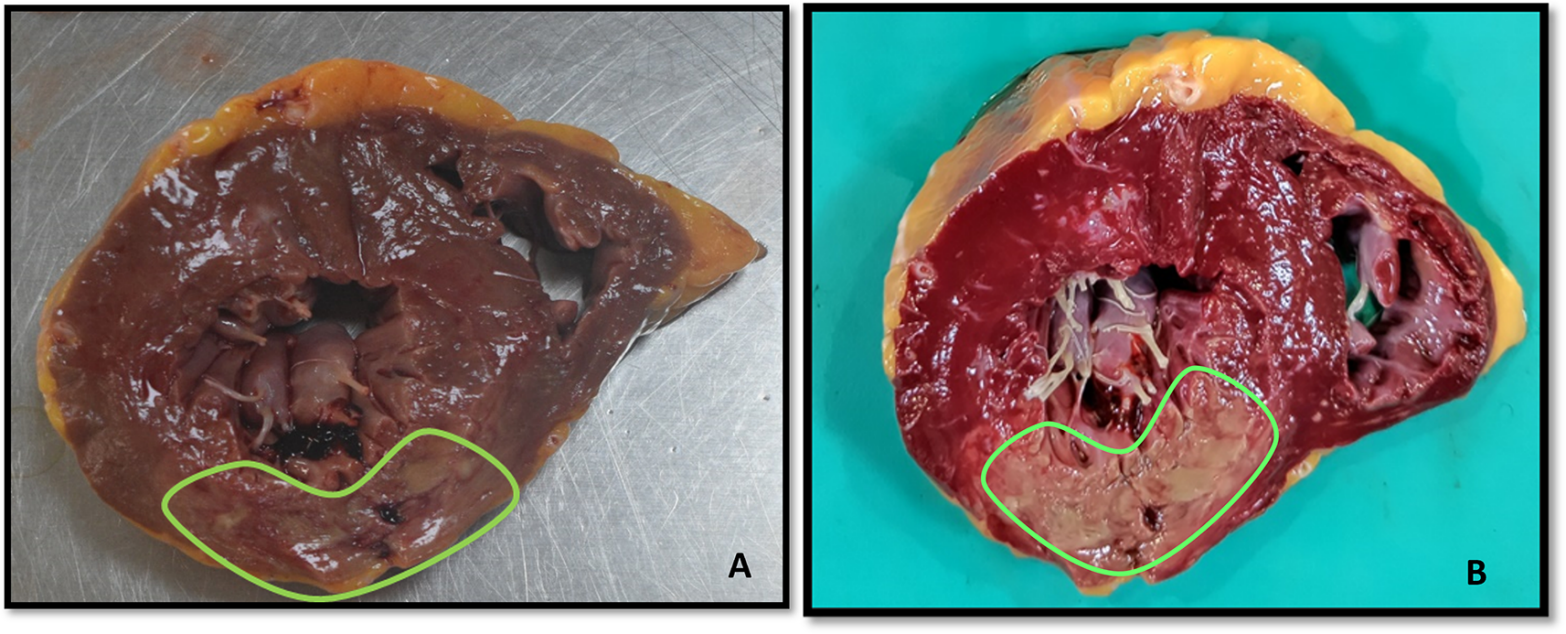
Section of heart showing transmural infarction (A. Before staining B. After staining).

**Figure 3.**
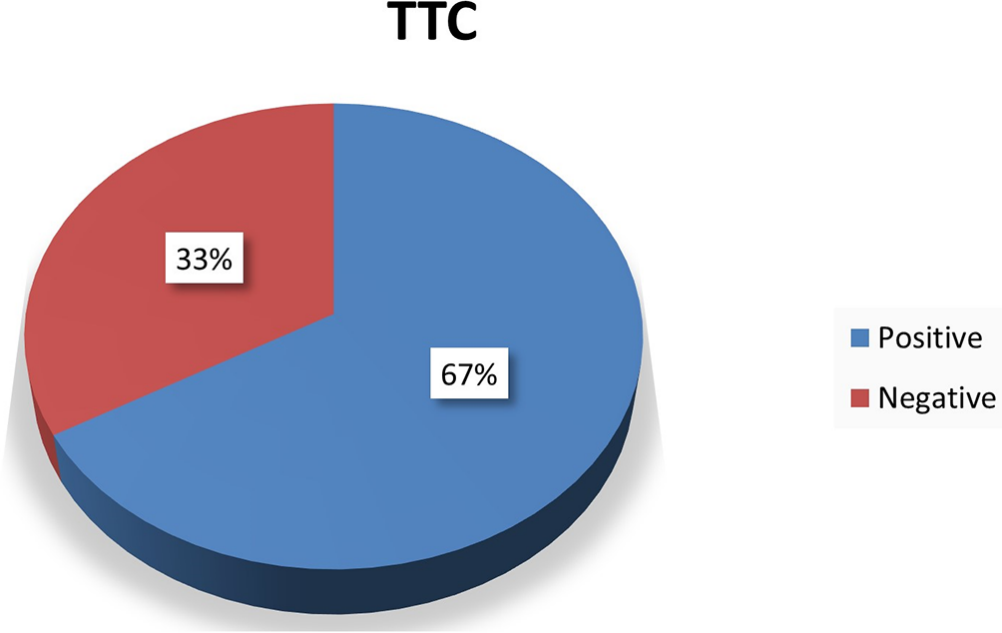
Chart showing TTC staining results.

The 60 hearts were stained with TTC out of which 40 hearts remained unstained suggestive of myocardial infarction and 20 hearts were stained suggestive of non-infarcted hearts ( Figure 2 and 3).

The Diagnostic evaluation of TTC with respective of HPE is shown in Table 3. The 60 hearts were stained with TTC out of which 40 hearts remained unstained suggestive of myocardial infarction and the unstained areas was subjected for histopathology examination to correlate the microscopic changes of MI, 34 hearts were diagnosed MI and validates the sensitivity of TTC test is 100% and rest 20 hearts were stained with TTC revealed non-infarcted heart and histopathology examination revealed negative for MI.

**Table 3.** Diagnostic evaluation of TTC with respective of HPE.

TTC	HPE		Total
	Positive	Negative	
Positive	34	6	40
	100.0%	23.1%	66.7%
Negative	0	20	20
	0.0%	76.9%	33.3%
Total	34	26	60
	100.0%	100.0%	100.0%

**Figure 4.**
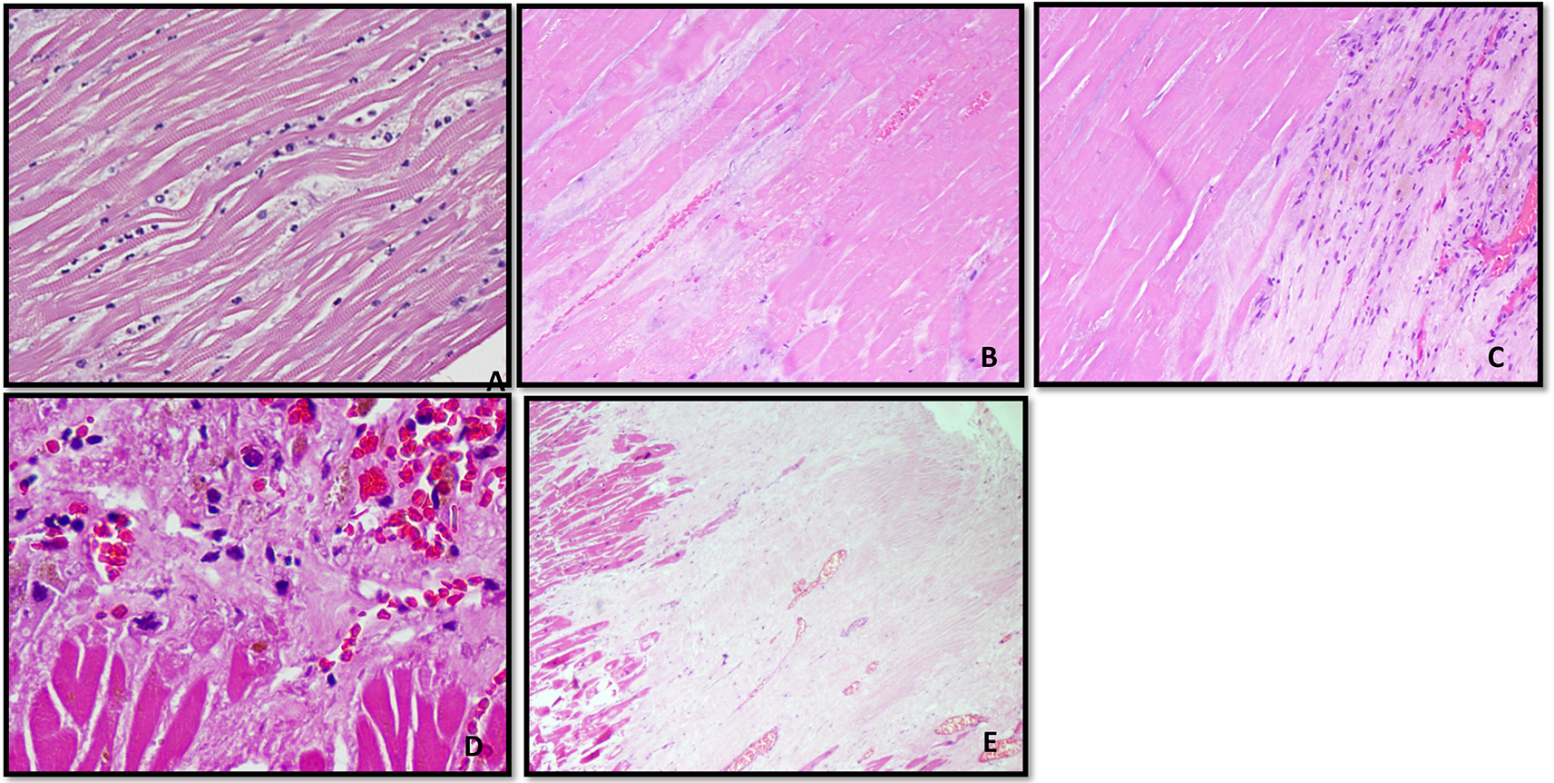
Changes in the histopathology of heart musculature based on time interval. A. Zone of an early infarct with waviness of the necrosed myocytes and few neutrophilic infiltrates (< 4 hours) (H&E, x100). B. Early infarct with coagulative necrosis of the cardiac myocytes (4-12 hours) (H&E, x200). C. Periphery of the infarct with early granulation tissue formation (7-10 days) (H&E, x100). D. Periphery of the infarct with organized granulation tissue formation (7-10 days) (H&E, x200). E. Fibrous scar with adjoining viable cardiac myocytes (> 6 weeks) (H&E, x200).

Changes in the histopathology of heart musculature based on time interval are shown in ( Figure 4). The zone of an early infarct with waviness of the necrosed myocytes and few neutrophilic infiltrates is seen in less than 4 hours. Early infarct with coagulative necrosis of the cardiac myocytes is seen in 4-12 hours. The periphery of the infarct with early and organized granulation tissue formation is seen in 7-10 days. Fibrous scar with adjoining viable cardiac myocytes is seen in more than 6 weeks. The time interval of myocardial estimation was estimated by subjecting the heart for histopathological examination is shown in Table 4. 51.7 % showed early infarction, 1.7 % showed late infarction, 5 % showed old myocardial infarction and 41.7 % revealed no infarction. Distribution of time interval of Myocardial infarction in HPE is shown in Table 5. Distribution of time interval of Myocardial infarction in TTC is shown in Table 6.

**Table 4.** Time interval of Myocardial infarction by HPE.

Age of myocardial infarction	Frequency	Percentage
No MI	25	41.7%
Early MI (4-12 hours)	31	51.7%
Late MI (3-7 days)	1	1.7%
Old MI (2-8 weeks)	3	5%
Total	60	100%

**Table 5.** Distribution of time interval of Myocardial infarction in HPE.

Time of MI	HPE		Total
	Positive	Negative	
No MI	0	25	25
	0.0%	96.2%	100.0%
Early MI	30	1	31
	88.2%	3.8%	100.0%
Late MI	1	0	1
	2.9%	0.0%	100.0%
Old MI	3	0	3
	8.8%	0.0%	100.0%
Total	33	27	60
	100.0%	100.0%	100.0%

**Table 6.** Distribution of time interval of Myocardial infarction in TTC.

Time of MI	TTC		Total
	Positive	Negative	
No MI	5	20	25
	20.0%	80.0%	100.0%
Early MI	31	0	31
	100.0%	0.0%	100.0%
Late MI	1	0	1
	100.0%	0.0%	100.0%
Old MI	3	0	3
	100.0%	0.0%	100.0%
Total	40	20	60
	66.7%	33.3%	100.0%

Accuracy of histopathological examination with TTC is shown in Table 7. It shows that TTC has an accuracy in diagnosing MI of 88.33%.

**Table 7.** Comparison of histopathological examination with TTC.

Statistic	Value	95% CI
Sensitivity	100.00%	89.42% to 100.00%
Specificity	74.07%	53.72% to 88.89%
Positive Likelihood Ratio	3.86	2.04 to 7.30
Negative Likelihood Ratio	0.00	
Disease prevalence *	55.00%	41.61% to 67.88%
Positive Predictive Value *	82.50%	71.36% to 89.92%
Negative Predictive Value *	100.00%	
Accuracy *	88.33%	77.43% to 95.18%

* These values are dependent on disease prevalence.

## Discussion

Acute myocardial infarction causes sudden cardiac mortality in both men and women. In many cases, death takes place rapidly, and post-mortem examination reveals no evidence of acute myocardial infarction. It manifests itself with substantial morphological alterations that take 24 to 48 hours to manifest. A random sectioning of the heart for Histopathological investigation is typically used to diagnose myocardial infarction. ^7^ Random cardiac sectioning for histopathological investigation is ineffective because it frequently misses myocardial infarction segment if it does not involve a larger infarct area. ^8^ To get around the difficulty, a variety of procedures have been used by forensic pathologists to confirm the diagnosis of acute MI. The heart is stained with triphenyl Tetrazolium chloride for early myocardial infarction is quicker and faster approach to use in the autopsy room and it can yield a reasonable outcome after the procedure. ^9^ Numerous animal studies used TTC staining for identifying acute myocardial infarction, but it might not be applicable to humans because of species-to-species differences. ^10^

Thunderchief et al, in 18 cases of sudden death, TTC staining technique was carried out in deceased heart and MI was more prevalent in male population the TTC staining test as 91.66 % sensitivity and 83.33% specificity. ^11^ M Shankar Bakkannavar et al, in 40 cases of sudden death, the TTC staining approach was conducted to do histochemical staining of the heart. The TTC staining approach has 100 % sensitivity and specificity, according to the study. Instead of phosphate buffer, hydrochloric acid is utilized to modify the pH of the solution in this study. ^12^

M Sivanandam conducted a study on 18 hearts taken from cases of sudden deaths with the cause of death thought to be of cardiac source. He found that TTC is a reliable method for the identification of early myocardial infarction during autopsy inspection with a diagnostic validity of 88.8%. ^13^ Adegboyega et al, used TTC dye to stain 638 hearts from individuals with suspected or confirmed MI. They found that TTC is a valuable adjuvant in detecting acute myocardial ischemia for histochemical identification of grossly inapparent Myocardial infarction. ^14^

Padole TO conducted a study on 107 hearts. He divided the heart into 2 groups 1) Control group which include cases of death due to cardiac sources. 2) Negative control group which include cases of sudden natural death due to non-cardiac sources. He found that in Group 1 there were 15 cases out of them in 80% of cases TTC staining technique was positive while in 33.33% of cases H&E method was positive. Similarly in Group 2, there were 92 cases, out of them in 43.47% cases TTC staining technique was positive and in 22.82% of cases H & E method was positive. This suggest that the TTC technique is more superior and precise technique to determine the cause of death as MI. ^15^ Abdulridha AA conducted a study in 75 cases of sudden natural death. And he exposed all the hearts to TTC solution staining and for histopathological evaluation for finding of a probable acute myocardial infarction. They found that after immersion in TTC solution, 45 heart samples exhibited macroscopic pale/yellow zones which signifies acute myocardial infarcts, while histopathological investigation of samples showed features of acute myocardial infarction in 62 samples. TTC stain was found to have 69.4% diagnostic sensitivity and 76.9% specificity in detection of acute myocardial infarction during autopsy. ^16^ Our study also shows that the Triphenyl Tetrazolium Chloride test is a reliable approach for forensic pathologists to diagnose early myocardial infarction during the post-mortem examination. It shows that TTC has an accuracy in diagnosing MI of 88.33%.

However, this approach has limitations in identifying the cause of death related to arrhythmic death when it occurs during the ischemia period before the onset of infarction. In such cases, the microscopic examination of cardiac tissue and conducting fibers will aid to arrive at the cause of death. However, the histochemical staining with TTC for detection of early stages of myocardial infarction can produce false positive and negative results. In numerous numbers of cases, a combination of histochemical method and histological evaluation aids in the diagnosis or exclusion of an apparent early myocardial infarction as the cause of death.

Limitations of the study:

1) The number of cases collected in this study is natural sudden death with history of cardiac illness.
2) Putrefaction alters the enzymes levels after death in such circumstances when heart is stained with TTC it can cause false positive results.

The result of this study shows that the Triphenyl Tetrazolium Chloride test, a histochemical staining technique of heart, is reliable approach for forensic pathologists to diagnose early myocardial infarction during the post-mortem examination. Preparing 1 % TTC solution and adjustment of pH phosphate buffers is rather easy. The procedure is also economical, easy to perform in a post-mortem hall and does not necessitate the use of complicated equipment. The method not only aids forensic pathologists in diagnosing early myocardial infarction, but it also aids general pathologists in determining where the sectioning should be performed to diagnose early myocardial infarction.

### Ethical and consent

Kasturba Medical College and Kasturba Hospital Institutional ethical clearance was taken before starting the study. Approval no with date: 705/2019, 17.9.2019.

Ethics committee has given the exemption from taking consent from the relatives of the deceased since these cases are postmortem cases where the law gives the consent for autopsy and determination of cause of death. In the above cases the samples were taken from death due to cardiac origin so, the heart must be taken out to determine the cause of death. The consent given by the law for autopsy is valid.

## Data Availability

Figshare: COMPARISION OF HISTOPATHOLOGICAL AND TRIPHENYL TETRAZOLIUM CHLORIDE TEST IN DIAGNOSING MYOCARDIAL INFARCTION: AN AUTOPSY STUDY. Doi: https://doi.org/10.6084/m9.figshare.25910422.v1. ^6^ This project contains the following underlying data:
- Research data.docx Data are available under the terms of the Creative Commons Attribution 4.0 International license (CC-BY 4.0). Figshare: We used STROBE checklist for “COMPARISION OF HISTOPATHOLOGICAL AND TRIPHENYL TETRAZOLIUM CHLORIDE TEST IN DIAGNOSING MYOCARDIAL INFARCTION: AN AUTOPSY STUDY”. DOI: https://doi.org/10.6084/m9.figshare.25910419.v1. ^4^ Data are available under the terms of the Creative Commons Attribution 4.0 International license (CC-BY 4.0).
