# Supplementary material for: Comparison of histopathological and triphenyl tetrazolium chloride test in diagnosing myocardial infarction: An autopsy study
Source: F1000Res. 2024 Sep 12;13:1050. [Version 1] doi: 10.12688/f1000research.152421.1 (PMC11411240; doi:10.12688/f1000research.152421.1)
Supplement: Peer review history

# Reviewer report: Reviewer response for version 1

**DOI:** 10.5256/f1000research.167177.r323365

**Published:** 2024-09-18

**Authors:** Mahabalesh Shetty

**Affiliations:** 1 FORENSIC MEDICINE, K S HEGDE MEDICAL ACADEMY ,NITTE (DEEMED TO BE UNIVERSITY ), Manglore, Karnataka, India

The article provides sufficient information about the methodology and analysis to enable others to allow replication.

 In addition, the analysis, experimentation, and article construction are all good. Statistical study confirms the author's claim that TTC detects early myocardial injury with significance and accuracy. The laboratory tests are essential in determining the Cause of Death in major cases of sudden natural death.

 The manuscript will aid in spreading knowledge about the significance of the TTC staining technique.

**The strength of the article:**

 The study can be conducted in any mortuary, which is the article's strength. Any expensive equipment is not required. There is a clear explanation of how to prepare TTC solution, and getting the ingredients is simple as they are readily available in the market. Both pathologists and forensic pathologists will benefit from the method's ability to identify myocardial infarction early in the morgue and during sectioning. 

**Weakness of the article:**

 A limitation of the research was the small number of cases—just 60 hearts—in the study.

 Putrefaction   alters  the  enzymes  levels  after death in such circumstances   when heart is  stained with TTC it can cause false positive results-This sentence is ambiguous .

 Putrefaction can be influenced by a wide range of variables and can start early under certain circumstances, which should have been highlighted.

 However, Reference number -12 in the article is, Shenoy et al  

 But the paper shows M Shankar Bakkannavar et al.

Is the work clearly and accurately presented and does it cite the current literature?

Yes

If applicable, is the statistical analysis and its interpretation appropriate?

Yes

Are all the source data underlying the results available to ensure full reproducibility?

Yes

Is the study design appropriate and is the work technically sound?

Yes

Are the conclusions drawn adequately supported by the results?

Yes

Are sufficient details of methods and analysis provided to allow replication by others?

Yes

Reviewer Expertise:

FORENSIC PATHOLOGIST .

I confirm that I have read this submission and believe that I have an appropriate level of expertise to confirm that it is of an acceptable scientific standard.

## References

[1] : Identification of Myocardial Infarction in Human Autopsy Population Using TTC. *JOURNAL OF PHARMACEUTICAL AND BIOMEDICAL SCIENCES* .2010;

# Reviewer report: Reviewer response for version 1

**DOI:** 10.5256/f1000research.167177.r323366

**Published:** 2024-09-17

**Authors:** Hareesh Gouda

**Affiliations:** 1 Fr Muller Medical College, Mangalore, Karnataka, India

Triphenyl Tetrazolium Chloride (TTC) staining method of the myocardial tissue is an important alternative or additional method to the routinely done Histo-pathological examination to find out early myocardial infarction during autopsy. Procedure of collection and  staining of the tissue has been explained in detail in the manuscript. And, also the TTC method is feasible to perform. Statistical analysis supports the claim made by the author about significance and accuracy of TTC method in detecting early myocardial damage. In significant cases of sudden natural death, the laboratory investigations play a vital role in the determination of Cause of Death. Indexing of the manuscript will help in disseminating the information about and importance of TTC staining method.

Is the work clearly and accurately presented and does it cite the current literature?

Yes

If applicable, is the statistical analysis and its interpretation appropriate?

Yes

Are all the source data underlying the results available to ensure full reproducibility?

Yes

Is the study design appropriate and is the work technically sound?

Yes

Are the conclusions drawn adequately supported by the results?

Yes

Are sufficient details of methods and analysis provided to allow replication by others?

Yes

Reviewer Expertise:

Forensic pathology, Toxicology

I confirm that I have read this submission and believe that I have an appropriate level of expertise to confirm that it is of an acceptable scientific standard.
